# Event-triggered adaptive predefined-time anti-unwinding attitude tracking control for spacecraft

**DOI:** 10.1371/journal.pone.0333700

**Published:** 2025-10-22

**Authors:** Guogang Wang, Zichao Feng, Youyang Qu, Hongwei Sun

**Affiliations:** 1 School of Information and Control Engineering, Jilin University of Chemical Technology, Jilin, China; 2 Laboratory of Attitude and Orbit Control, Chang Guang Satellite Technology CO.LTD, Changchun City, China; University of Shanghai for Science and Technology, CHINA

## Abstract

This paper addresses the problem of predefined-time attitude tracking control for rigid spacecraft subject to external disturbances and unknown inertia parameters. First, a predefined-time non-singular sliding surface is designed to ensure that the closed-loop system converges within a predefined time. Second, to tackle the unwinding problem inherent in quaternion-based modeling, a potential function is introduced in the controller design to guarantee anti-unwinding performance even outside the sliding surface. The proposed controller not only suppresses chattering but also ensures both anti-unwinding behavior and predefined-time convergence. Then, an event-triggered mechanism is developed to reduce communication burden while avoiding Zeno behavior. The proposed control method can make the attitude tracking error converge to an arbitrary predefined residual set. Finally, simulation results verify the effectiveness of the proposed method.

## 1. Introduction

Spacecraft attitude control technology can be used in many missions such as satellite communication, ocean monitoring and weather forecasting, but spacecraft operating in complex environments often face parametric uncertainties and external disturbances. To address these challenges, many control strategies for spacecraft attitude control have been developed such as sliding mode control [1,2], optimal control [3,4], event-triggered control [5,6], model predictive control [7,8], and backstepping control [9,10].

Sliding mode control (SMC) is a very efficient method of handling parameter uncertainties and external disturbances, and hence has been used extensively to design spacecraft attitude controllers. Finite time and fixed time sliding mode attitude tracking control schemes are proposed for rigid spacecraft, however the linear sliding mode control has certain drawbacks, such as the chattering on the sliding surface [11]. To overcome these issues, many researchers have investigated nonlinear terminal sliding mode control schemes to achieve faster convergence and better dynamic performance. Song and Li [12] proposed finite time fast terminal sliding mode control scheme with dual loop structure for spacecraft attitude control. Chen et al. [13] considered modeling uncertainties, external disturbances and actuator saturation. By combining fast terminal sliding mode surface (FTSMS) and low-pass filter, they developed novel integral terminal sliding mode surface (ITSMS) that converge fast finite-time convergence of the control system. However, these methods did not address singularity problem inherent in spacecraft dynamics. Zheng et al. [14] constructed nonsingular terminal sliding mode surfaces to improve the accuracy of attitude tracking control of rigid spacecraft. A nonsingular controller is proposed in literature, which guarantees that the closed-loop system is almost globally stable in fixed time (i.e., all initial states converge to equilibrium point in fixed time except zero measure set). Gao et al. [15] designed a jitter-free fault-tolerant controller based on nonsingular terminal sliding mode (NTSM), which provides a new solution for finite time attitude maneuver of rigid spacecraft. Alipour [16] proposed a new adaptive fractional order nonsingular terminal sliding mode (AFONTSM) controller, and studied the fixed-time stability of closed-loop systems under uncertainties and external disturbances by using Lyapunov theorem.

When quaternion is used to represent the attitude of spacecraft, there are two target values in quaternion scalars. If one of them is ignored artificially, the spacecraft will need to rotate an angle greater than 180 to complete the task when it can rotate at a small angle (called unwinding phenomenon), resulting in unnecessary energy waste. Therefore, it is necessary to avoid unwinding phenomenon in the process of designing attitude controller. To overcome this problem, Dong and Wu et al. [17] designed a terminal sliding mode control law with hyperbolic sine switching function, which solved buffeting by introducing boundary layer and gave a subset of attraction domain of equilibrium point, so as to realize finite time convergence and attitude tracking without unwinding. Su and Xu et al. [18] designed a preset-time sliding mode disturbance observer to estimate the disturbance, and constructed a new sliding mode surface with two equilibrium points by introducing a hyperbolic sine function to ensure the preset-time convergence and avoid unwinding phenomenon. Muhammad Amrr [19] proposed a finite-time composite control method to solve external disturbances, actuator failures and quaternion unwinding problems, Huang and Li et al. [20] proposed two anti-unwinding control schemes to ensure that the spacecraft attitude tracks the desired attitude in finite time. The first controller is designed by a new sliding mode surface without considering external disturbances and gives a convergence time expression. The second controller can achieve fixed-time stability in the presence of external disturbances by modifying the sliding mode surface and introducing adaptive laws. Guang and Li et al. [21] designed a nonsingular fixed-time sliding mode controller, which achieved fixed-time convergence and anti-unwinding characteristics. Zuo et al. [22] proposed a novel observer-based fixed-time control technique to achieve system stabilization.

In recent years, researchers have focused on predefined-time control algorithms [23–26]. Liu et al. [27] constructed a composite barrier Lyapunov function to avoid the unwinding phenomenon during spacecraft attitude control. Based on a new time-scale transformation of the attitude system, they designed an adaptive backstepping attitude controller to ensure stable performance. Xu et al. [28] addressed the problem of anti-unwinding attitude stabilization for rigid spacecraft by proposing a predefined-time adaptive sliding mode surface. The proposed method guarantees both predefined-time convergence and anti-unwinding performance. Wang et al. [29]addressed the control problem with input constraints by developing an algorithm that achieves consensus while strictly satisfying predefined amplitude and rate limitations.

Modern control systems are increasingly implemented over communication networks, which offer cost reduction and flexibility advantages. However, because of limited bandwidth of such networks, event-triggered control has been proposed and attracting increasing interest. In this control model, controller communicates with actuators or sensors only at discrete time events when a predefined trigger condition is met, and thus the communication load is reduced. Wang et al. [30] proposed an event-triggered adaptive backstepping controller using switching threshold strategy to construct the event-triggering mechanism. This compensates for network errors and disturbances and avoid Zeno phenomena. Wang et al. [31] proposed a predefined time event-triggered sliding mode attitude synchronization strategy for distributed multi-spacecraft systems. Wang and Zuo [32]introduced a new distributed event-triggered algorithm, in which control updates are executed only when specific triggering conditions are satisfied. They rigorously proved the existence of a strictly positive minimum inter-event time, thereby excluding the occurrence of Zeno behavior.

The main contributions of this paper are as follows:

1. Compared with [17] and [21], this paper considers the effects of inertia uncertainties and unknown external disturbances, as well as the communication burden of spacecraft. The proposed predefined-time controller demonstrates superior convergence performance under these more realistic conditions.
2. Unlike [27] and [28], this paper designs a predefined-time non-singular sliding surface that guarantees strict predefined-time convergence and anti-unwinding performance on the surface. Moreover, a potential function is introduced to design the controller, ensuring anti-unwinding behavior even outside the sliding surface. The attitude tracking error is proven to converge to an arbitrarily small residual set within the predefined time.
3. Noting that most existing anti-unwinding attitude tracking control studies neglect communication constraints, this paper proposes a predefined-time adaptive event-triggered anti-unwinding control strategy, which effectively reduces communication burden while avoiding the unwinding phenomenon.

The rest of the paper is structured as follows:

Section 2 presents the relative kinematic and dynamic models of the rigid spacecraft, introduces the unwinding phenomenon, and states the control objectives. Section 3 details the controller design. Section 4 provides simulation results. Section 5 concludes the paper.

## 2. Preliminaries and problem formulation

### 2.1 Attitude tracking error kinematics and dynamics

**Fig 1:**
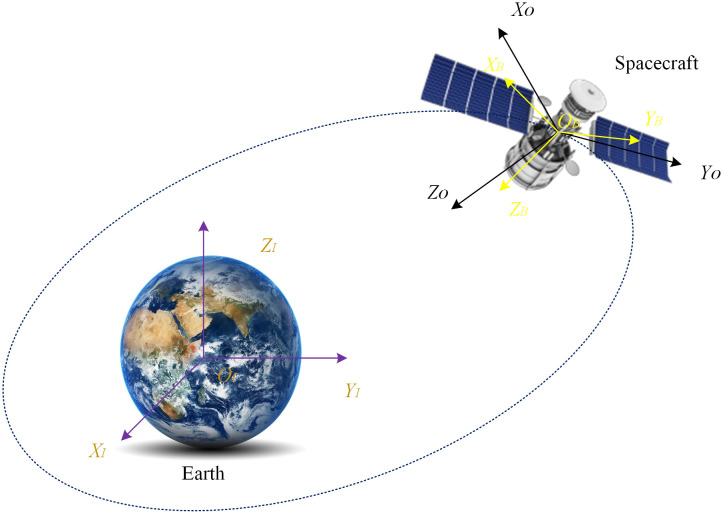
Schematic of Spacecraft Motion.

The selection of reference coordinate frames is illustrated in Fig 1. The inertial coordinate frame is denoted as $I(O_{I}-X_{I}Y_{I}Z_{I})$, the orbital coordinate frame as $O(O-X_{B}Y_{B}Z_{B}\backslash )$, and the spacecraft body-fixed frame as $O(O-X_{O}Y_{O}Z_{O}$).

The spacecraft’s attitude is defined by the orientation of the body-fixed axes.

$X_{O}$,$Y_{O}$ and $Z_{O}$ with respect to the reference frame. While position vectors are expressed using the three axes of the reference frame, attitude parameters are described using a unit quaternion representation.

When the external disturbances and inertia uncertainty are taken into account, the attitude dynamic equation of a rigid spacecraft is described as [33]


$$\begin{array}{c} \dot{q}=\frac{\text{1}}{\text{2}}[\begin{array}{c} -{q_{v}}^{\text{T}}\\ q_{\text{0}}+I_{\text{3}}+{q_{v}}^{\times } \end{array}]\omega \\ J\dot{\omega }=-\omega^{\times }J\omega +u+d \end{array}$$ (1)

Where $I_{\text{3}}$ represents the 3 × 3 identity matrix; The unit quaternion $q={\left[q_{\text{0}}{q_{v}}^{\text{T}}\right]}^{\text{T}}\in R^{\text{3}}$ represents the spacecraft’s attitude, $q_{\text{0}}$ is the initial attitude, $q_{v}={\left[q_{\text{1}},q_{\text{2}},q_{\text{3}},\right]}^{T}$. The spacecraft’s angular velocity is denoted as $\omega ={\left[\omega_{\text{1}}\omega_{\text{2}}\omega_{\text{3}}\right]}^{\text{T}}\in R^{\text{3}}$, $\omega =\left[\begin{array}{ccc} 0 & -\omega_{3} & \omega_{2}\\ \omega_{3} & 0 & -\omega_{1}\\ -\omega_{2} & \omega_{1} & 0 \end{array}\right]$.The matrix $J=J_{0}+\Delta J\in R^{\text{3}\times \text{3}}$ represents total inertia of the spacecraft, and $J_{\text{0}}$ and ΔJ are the nominal and uncertainty part of the inertia, respectively. $u\in R^{\text{3}}$ is the control torque. $d\in {\mathbb{R}}^{\text{3}}$ is an unknown external disturbance. $q_{d}={\left[q_{d\text{0}}\;q_{dv}^{\text{T}}\right]}^{\text{T}}\in {\mathbb{R}}^{\text{3}}$ denote the desired attitude quaternion, and $\;\omega_{d}={\left[\omega_{d\text{1}}{\;\omega }_{d\text{2}}{\;\omega }_{d\text{3}}\right]}^{\text{T}}\in {\mathbb{R}}^{\text{3}}$ denote the desired angular velocity.

Let $q_{e}={\left[q_{e\text{0}}\;q_{ev}^{\text{T}}\right]}^{\text{T}}\in {\mathbb{R}}^{\text{3}}$ denote the attitude tracking error, where $q_{ev}={\left[q_{\text{ev1}}\;q_{\text{ev2}}\;q_{\text{ev3}}\;\right]}^{\text{T}}\in {\mathbb{R}}^{\text{3}}$. Since $q_{e}\in {\mathbb{R}}^{\text{3}}$, then there holds $q_{ev}^{\text{T}}q_{ev}+q_{e\text{0}}^{\text{2}}=\text{1}$.

Besides, we know [34]


$$\left\{ \begin{array}{c} q_{\text{e0}}=q_{\text{dv}}^{T}{\text{q}}_{\text{v}}+q_{\text{d0}}q_{\text{0}}\;\;\;\;\;\;\;\\ q_{\text{ev}\;=}q_{\text{d0}}q_{\text{v}}-q_{\text{dv}}^{\times }q_{\text{v}}-q_{\text{0}}q_{\text{dv}} \end{array}\right.$$ (2)

The kinematics equation of the error system is described as


$${\dot{q}}_{e}=\frac{\text{1}}{\text{2}}[\begin{array}{c} -q_{ev}^{T}\\ q_{e0}I_{3}+q_{ev}^{\times } \end{array}]\omega_{e}$$ (3)

Let $\omega_{e}\in {\mathbb{R}}^{\text{3}}$ denote the attitude angular velocity tracking error. Then, we have


$$\omega_{e}=\omega -G\omega_{d}$$ (4)

Where G is the rotation matrix defined by


$$G=\left(q_{e\text{0}}^{\text{2}}-q_{ev}^{T}q_{ev}\right)I_{\text{3}}+\text{2}q_{ev}q_{ev}^{T}-2q_{e\text{0}}q_{ev}^{\times }$$ (5)

The time derivative of $\omega_{e}$ is given by


$${\dot{\omega }}_{e}=\dot{\omega }+\omega_{e}^{\times }G\omega_{d}-G{\dot{\omega }}_{d}$$ (6)

In summary, the error dynamics model of the spacecraft attitude tracking system can be written as follows [37]


$$\left\{ \begin{array}{c} {\dot{q}}_{e\text{0}}=-\frac{\text{1}}{\text{2}}q_{ev}^{T}\omega_{e}\;\;\;\;\;\;\\ {\dot{q}}_{ev}=\frac{\text{1}}{\text{2}}(q_{e\text{0}}I_{\text{3}}+q_{ev}^{\times })\omega_{e} \end{array}\right.$$ (7)

$$J{\dot{\omega }}_{e}=-(\omega_{e}+G\omega_{d}{)}^{\times }J\left(\omega_{e}+{G\omega }_{d}\right)+J\left(\omega_{e}^{\times }G\omega_{d}-G\dot{\omega_{d}}\right)+u+d$$ (8)

By combining with equation (1), equation (8) can be rewritten as


$$J{\dot{\omega }}_{e}=-(\omega_{e}+G\omega_{d}{)}^{\times }J_{\text{0}}\left(\omega_{e}+{G\omega }_{d}\right)+J_{\text{0}}\left(\omega_{e}^{\times }G\omega_{d}-G\dot{\omega_{d}}\right)+u+d_{\text{1}}$$ (9)

with the residual dynamics $d_{\text{1}}$ is defined by


$$d_{\text{1}}=-(\omega_{e}+G\omega_{d}{)}^{\times }\Delta J\left(\omega_{e}+{G\omega }_{d}\right)+\Delta J\left(\omega_{e}^{\times }G\omega_{d}-G\dot{\omega_{d}}\right)+d$$ (10)

In this article, we suppose that the following assumptions hold

**Assumption 1**

It is assumed that there exists an unknown upper bound for the external disturbance d

**Assumption 2**

ΔJ and its derivative $\Delta \dot{J}$ and have upper bounds.

**Assumption 3**

The initial attitude error of the spacecraft satisfies $q_{e\text{0}}(t_{s}neq\text{0}.$

**Remark1**

Generally, the sources which emanate the disturbances in the spacecraft are from aerodynamic drag, magnetic forces, pressure due to solar radiation, gravitation, etc.

All these sources of disturbances are assumed bounded.This is an explanation for Assumption 1

Assumption 2 is essential. It reflects the reality of inertial parameter uncertainties in spacecraft, ensure the stability and feasibility of control algorithms when dealing with such uncertainties, and facilitate theoretical analysis by providing clear bounds for stability proofs and convergence derivations, making the research both practical and theoretically rigorous.

**Remark2**

Assumption 3 is introduced to ensure that the potential function does not become singular at the initial stage. Specifically, if $q_{e}=\text{0}$ at the beginning, the function would be singular, and once this singularity occurs, the anti-unwinding control strategy can no longer be applied.

### 2.2 Unwinding phenomenon

According to Euler’s rotation theorem, the problem of spacecraft attitude change can be described using Euler axis-angle representation. The spacecraft rotates about the Euler axis $\text{e}\in {\mathbb{R}}^{\text{3}}$, with a rotation angle $\;\theta (tin[\begin{array}{cc} 0 & 2\pi \end{array}]$, transitioning from the initial attitude $q_{b}$ to the desired attitude $q_{d}$.

Therefore, based on Euler’s rotation theorem, the attitude error quaternion $q_{e}$ representing the transition from the current attitude $q_{b}$ to the desired attitude $q_{d}$ can be expressed as follows [18].


$$q_{e}=[\begin{array}{c} q_{e\text{0}}\\ q_{ev} \end{array}]=[\begin{array}{c} \text{cos}\frac{\theta (t)}{\text{2}}\\ e\text{sin}\frac{\theta (t)}{\text{2}} \end{array}]$$ (11)

Denote


$$\begin{array}{c} \begin{array}{c} \eta_{\text{1}}=\left\{{(q}_{e},\omega_{e})\in S^{\text{3}}\times R^{\text{3}}|q_{e}=[\text{1000}{]}^{\text{T}},\omega_{e}=\text{0}\right\},\\ \eta_{\text{2}}=\left\{{(q}_{e},\omega_{e})\in S^{\text{3}}\times R^{\text{3}}|q_{e}=[-\text{1000}{]}^{\text{T}},\omega_{e}=\text{0}\right\} \end{array} \end{array}$$ (12)

In the physical attitude rotation space, the attitudes represented by θ(t)=2kπ(k=0,2,4···) and θ(t)=2kπ(k=1,3,5···) are actually identical. According to equation (10), when k=0,2,4··· or k=1,3,5···,it follows that ${\;q_{e\text{0}}|}_{\theta (t)=\text{2}k\pi }=\text{1}$ or ${\;q_{e\text{0}}|}_{\theta (t)=\text{2}k\pi }=-\text{1}$,and when k=0,1,2... ${\;q_{ev}|}_{\theta (t)=\text{2}k\pi }=\text{0}$. Therefore,$\;\eta_{\text{1}}$

and $\eta_{\text{2}}$ are two equilibrium points of the spacecraft attitude error dynamics described in equation (6). In this case, if the initial value of −1, then $q_{e}$ will change from −1 to 1, which means that despite the spacecraft’s initial attitude being the same as the desired attitude, the spacecraft must still rotate 2π to reach the desired state. This is known as the unwinding phenomenon.

### 2.3 Control goal

The objective of this study is to design a controller that enables attitude tracking in the presence of disturbances while also exhibiting anti-unwinding characteristics. A control law needs to be developed for system equations (6) and (7), which must satisfy the following conditions


$$\left\{ \begin{array}{c} \lim_{t\to T}q_{e}\to q_{I}\;\;\;\;\;\;\{q_{e0}(t)>0\;\forall_{t}\in [t_{0},T],\;\;\;if\;q_{e0}(t_{0})>0\\ \lim_{t\to T}q_{e}\to -q_{I}\;\;\;\{q_{e0}(t)<0\;\forall_{t}\in [t_{0},T],\;\;if\;q_{e0}(t_{0})<0 \end{array}\right.$$ (13)

**Lemma 1** [35]. For x∈Rand ϵ≥0,one can obtain


$$\text{0}\leq |x|\leq \sqrt{\epsilon }+\frac{x^{\text{2}}}{\sqrt{x^{\text{2}}+\epsilon }}$$ (14)

**Lemma 2** [36]. Consider the following system.


$$\dot{x}=f\left(x,t\right)\;\;\;f\left(0,t\right)=\text{0}\;\;\;x\in R^{n}$$ (15)

where $f\to R^{n}\times R^{+}\to R^{n}$ is a continuous function. The initial condition of the system is defined as $x(\text{0})=x_{\text{0}}$. If there exists a Lyapunov function V defined on $R^{n}\times R^{+}$ that satisfies the following inequality


$$\dot{V}\leq -\frac{\pi }{\alpha T_{c}\sqrt{mn}}(mV^{\text{1}-\frac{\alpha }{\text{2}}}+nV^{\text{1}+\frac{\alpha }{\text{2}}}+\text{2}\sqrt{mnV})$$ (16)

**Lemma 3** [36].For the system $\dot{x}=f(x,t)$, if there exists a Lyapunov function defined on $R^{n}\times R^{+}$, satisfying the following inequality


$$\dot{V}\left(x,t\right)\leq -\frac{\pi }{\alpha T_{p}\sqrt{mn}}\left(mV^{\text{1}-\frac{\alpha }{\text{2}}}\left(x,t\right)+nV^{\text{1}+\frac{\alpha }{\text{2}}}\left(x,t\right)+\text{2}\sqrt{mnV}\right)+\vartheta$$ (17)

where 0<α<1, $T_{p}>\text{0},m>\text{0},n>\text{0}$, are predefined positive constants, and ϑ is bounded. then the system’s convergence time and convergence region satisfy the following relation


$$\left\{\underset{t\to T_{p}^{\prime }}{\text{lim}}\;x|\;V(x)\leq \text{min}\left\{{\left(\frac{\text{2}\vartheta \alpha \sqrt{mn}}{n\pi }\right)}^{\frac{\text{2}}{\text{2}-\alpha }},{\left(\frac{\text{2}\vartheta \alpha \sqrt{mn}}{n\pi }\right)}^{\frac{\text{2}}{\text{2}+\alpha }}\right\}\right\}$$ (18)

where $T_{p}^{\prime }<T_{\text{max}}=\sqrt{\text{2}T_{p}}$. It is worth noting that $T_{\text{max}}$ is bounded.

**Lemma 4** [37]. For all positive numbers $x_{i}(i=\text{1},\text{2},\text{3},\ldots ,n)$ and v>0, one can obtain


$$\begin{array}{c} \left\{ \begin{array}{c} \sum_{i=\text{1}}^{n}x_{i}^{v}\geq (\sum_{i=\text{1}}^{n}x_{i}{)}^{v},\;\;\;\;\;\text{if}\;\text{0}<v<\text{1}\\ \sum_{i=\text{1}}^{n}x_{i}^{v}\geq n^{\text{1}-v}(\sum_{i=\text{1}}^{n}x_{i}{)}^{v},\;\;\text{if}\;v>\text{1}\;\;\;\; \end{array}\right. \end{array}$$ (19)

## 3. Anti-unwinding attitude tracking control

### 3.1 Non-singular predefined-time sliding surface

To achieve attitude tracking control of the spacecraft, a non-singular predefined-time sliding surface is designed as follows


$$S=\omega_{e}+\text{sign}(q_{e\text{0}})\gamma q_{ev}$$ (20)

where


$$\gamma =\frac{\text{2}\pi }{\alpha_{\text{1}}T_{\text{1}}\sqrt{a_{\text{1}}b_{\text{1}}}}\left\{ \begin{array}{c} \left(a_{\text{1}}\rho \text{1}-\text{0}.\text{5}\alpha_{\text{1}}+b_{\text{1}}\rho \text{1}\text{0}.\text{5}\alpha_{\text{1}}+\text{2}\sqrt{a_{\text{1}}b_{\text{1}}}\right),\;\;\;\text{if}\;\rho_{\text{1}}\geq \varepsilon \\ l_{\text{1}}\rho_{\text{1}}+l_{\text{2}}\rho \text{12}+\text{2}\sqrt{a_{\text{1}}b_{\text{1}}},\;\;\;\text{if}\;\rho_{\text{1}}<\varepsilon \end{array}\right.$$ (21)

where $\text{0}<\alpha_{\text{1}}<\text{1},T_{\text{1}}>\text{0},a_{\text{1}}>\text{0},b_{\text{1}}>\text{0},\rho_{\text{1}}=q_{ev}^{⊺}q_{ev}$,ε>0

where $\dot{\gamma }$ is given as


$$\dot{\gamma }=\frac{\text{2}\pi }{\alpha_{\text{1}}T_{\text{1}}\sqrt{a_{\text{1}}b_{\text{1}}}}\left\{ \begin{array}{c} \left({-a}_{\text{1}}{\alpha_{\text{1}}\rho }_{\text{1}}^{-\text{0}.\text{5}\alpha_{\text{1}}-\text{1}}q_{ev}^{⊺}{\dot{q}}_{ev}+b_{\text{1}}\alpha_{\text{1}}\rho_{\text{1}}^{\text{0}.\text{5}\alpha_{\text{1}}-\text{1}}q_{ev}^{⊺}{\dot{q}}_{ev}\right),\;\;\;\text{if}\;\rho_{\text{1}}\geq \varepsilon \\ {\text{2}l}_{\text{1}}q_{ev}^{⊺}{\dot{q}}_{ev}+\text{2}l_{\text{2}}{\rho_{\text{1}}q}_{ev}^{⊺}{\dot{q}}_{ev},\;\;\;\;\;\text{if}\;\rho_{\text{1}}<\varepsilon \end{array}\right.$$ (22)

Considering the arguments in (21) and (22), it is sufficient to ensure that both γ and $\dot{\gamma }$ are consistent when ${\rho_{\text{1}}=q}_{ev}^{⊺}q_{ev}$.The continuity of γ and $\dot{\gamma }$ can be guaranteed by appropriately choosing their values, ensuring they remain well-defined under the given conditions.

$\underset{\rho \to \varepsilon^{+}}{\text{lim}\;}\gamma =\underset{\rho \to \varepsilon^{-}}{\text{lim}}\gamma =\left(a_{\text{1}}\rho_{\text{1}}^{-\text{0}.\text{5}\alpha_{\text{1}}}+b_{\text{1}}\rho_{\text{1}}^{\text{0}.\text{5}\alpha_{\text{1}}}+\text{2}\sqrt{a_{\text{1}}b_{\text{1}}}\right)$ and $\underset{\rho \to \varepsilon^{+}}{\text{lim}}\dot{\gamma }=\underset{\rho \to \varepsilon^{-}}{\text{lim}}\dot{\gamma }=\left({-a}_{\text{1}}{\alpha_{\text{1}}\rho }_{\text{1}}^{-\text{0}.\text{5}\alpha_{\text{1}}-\text{1}}q_{ev}^{⊺}{\dot{q}}_{ev}+b_{\text{1}}\alpha_{\text{1}}\rho_{\text{1}}^{\text{0}.\text{5}\alpha_{\text{1}}-\text{1}}q_{ev}^{⊺}{\dot{q}}_{ev}\right)$

The parameters $l_{\text{1}}$ and $l_{\text{2}}$ must satisfy the following relationship


$$\left\{ \begin{array}{c} l_{\text{1}}\varepsilon +l_{2}\varepsilon^{\text{2}}=a_{\text{1}}\varepsilon^{-\frac{\alpha_{\text{1}}}{\text{2}}}+b_{\text{1}}\varepsilon^{\frac{\alpha_{\text{1}}}{\text{2}}}\;\;\;\;\;\;\\ l_{\text{1}}+\text{2}l_{\text{2}}\varepsilon =-\frac{a_{\text{1}\alpha_{\text{1}}}}{\text{2}}\varepsilon^{-\frac{\alpha_{\text{1}}}{\text{2}}-\text{1}}+\frac{b_{\text{1}\alpha_{\text{1}}}}{\text{2}}\varepsilon^{\frac{\alpha_{\text{1}}}{\text{2}}-\text{1}} \end{array}\right.$$ (23)

By solving the above system of equations, we obtain


$$\left\{ \begin{array}{c} l_{\text{1}}=\left({\text{2}a}_{\text{1}}+\frac{a_{\text{1}}\alpha_{\text{1}}}{\text{2}}\right)\varepsilon^{-\frac{\alpha_{\text{1}}}{\text{2}}-\text{1}}+\left({\text{2}b}_{\text{1}}-\frac{b_{\text{1}}\alpha_{\text{1}}}{\text{2}}\right)\varepsilon^{\frac{\alpha_{\text{1}}}{\text{2}}-\text{1}}\\ l_{\text{2}}=\left(-\frac{a_{\text{1}}\alpha_{\text{1}}}{\text{2}}-a_{\text{1}}\right)\varepsilon^{-\frac{\alpha_{\text{1}}}{\text{2}}-\text{2}}+\left(\frac{b_{\text{1}}\alpha_{\text{1}}}{\text{2}}-b_{\text{1}}\right)\varepsilon^{\frac{\alpha_{\text{1}}}{\text{2}}-\text{2}} \end{array}\right.$$ (24)

The continuity of $l_{\text{1}}$ and $l_{\text{2}}$ can be guaranteed by the aforementioned equations (23) and (24); therefore, the continuity of the sliding mode surface can be ensured through the above-mentioned method.

Here, γ acts as a dynamic adjustment parameter associated with the tracking attitude error ρ. Since the sliding mode incorporates a predefined time T and must ensure the continuity of the sliding surface, the continuity of its derivatives is guaranteed.

**Theorem 1.** the unwinding-free performance and the Predefined-time convergence property of the prosed sliding mode function (20) are ensured by

the theorem. If (6) is constrained to the sliding surface S=0, the following can be deduced


$$\left\{ \begin{array}{c} \underset{t\to T_{\text{1}}}{\text{lim}}∥q_{e}-q_{I}∥\leq \sqrt{\varepsilon }\;\;\;\{q_{e\text{0}}(t)>\text{0}\;\;\forall t\in [t_{s},T_{\text{1}}]\},\;\text{if }q_{e\text{0}}(t_{s})>\text{0}\\ \underset{t\to T_{\text{1}}}{\text{lim}}∥q_{e}+q_{I}∥\leq \sqrt{\varepsilon }\;\;\;\{q_{e\text{0}}(t)>\text{0}\;\;\forall t\in [t_{s},T_{\text{1}}]\},\;\text{if }q_{e\text{0}}(t_{s})<\text{0} \end{array}\right.$$ (25)

where, $\;t_{s}$ is the instant at which the attitude trajectory attains the sliding surface S=0. $\;T_{\text{1}}$ is the predefined time for the attitude to reach the equilibrium point.

**Proof**

First, it is proved that the sliding mode has anti-unwinding property in the sliding mode plane. Second, it is proved that the attitude error converges with predefined time.

The proof of anti-unwinding behavior needs to be proved in two cases, namely $q_{e\text{0}}\left(t_{s}\right)<\text{0}$ and $q_{e\text{0}}(t_{s})>0$. We only choose $q_{e\text{0}}(t_{s})>\text{0}$ to prove it.

when $\rho_{\text{1}}\geq \varepsilon$, $\|q_{ev}\|\geq \epsilon$, combining (6), (18), and (19)


$${\dot{q}}_{e\text{0}}=-\frac{\text{1}}{\text{2}}q_{ev}^{T}\omega_{e}=\frac{\pi }{\alpha_{\text{1}}T_{\text{1}}\sqrt{a_{\text{1}}b_{\text{1}}}}(a_{\text{1}}\rho_{\text{1}}^{\text{1}-\text{0}.\text{5}\alpha_{\text{1}}}+b_{\text{1}}\rho_{\text{1}}^{\text{1}+\text{0}.\text{5}\alpha_{\text{1}}}+\text{2}\sqrt{a_{\text{1}}b_{\text{1}}\rho_{\text{1}}})\geq \text{0}$$ (26)

when $\rho_{\text{1}}<\varepsilon$, $\|q_{ev}\|<\epsilon$,we can get


$${\dot{q}}_{e\text{0}}=-\frac{\text{1}}{\text{2}}qevT\omega_{e}=\frac{\pi }{\alpha_{\text{1}}T_{\text{1}}\sqrt{a_{\text{1}}b_{\text{1}}}}(l_{\text{1}}\rho_{\text{1}}+l_{\text{2}}\rho \text{12}+\text{2}\sqrt{a_{\text{1}}b_{\text{1}}})qevTq_{ev}\geq \text{0}$$ (27)

According to equations (24) and (25), it can be concluded that when $q_{e\text{0}}(t_{s})>\text{0}$, $\;{\dot{q}}_{e\text{0}}$ is always nonnegative, and the sign invariance is preserved. Therefore, once the sliding surface s=0, the designed sliding surface ensures that the spacecraft exhibits anti-unwinding behavior and avoids unnecessary attitude tracking control.

We only provide a proof for the case where $q_{e\text{0}}(t_{s})>\text{0}$, a similar method can be used to prove the case where $q_{e\text{0}}\left(t_{s}\right)<\text{0}$.

In order to prove the predefined-time convergence of $q_{e}$, we choose $V_{\text{1}}$ as the Lyapunov function, given by


$$V_{\text{1}}=\frac{\text{1}}{\text{2}}{\left(q_{ev}^{T}q_{ev}+(\text{1}-\text{sign}(q_{e\text{0}})q_{e\text{0}}\right)}^{\text{2}})$$ (28)

Given that the spacecraft’s attitude error quaternion satisfies $V_{\text{1}}=\frac{\text{1}}{\text{2}}q_{e}^{T}q_{e}$, from (26), we obtain


$$V_{\text{1}}=\text{1}-\left|q_{e\text{0}}\right|{\leq q}_{ev}^{T}q_{ev}<\text{1}$$ (29)

In view of (29), one has


$$\begin{array}{c} \left(a_{\text{1}}\rho \text{1}\text{1}-\text{0}.\text{5}\alpha_{\text{1}}+b_{\text{1}}\rho \text{1}\text{1}+\text{0}.\text{5}\alpha_{\text{1}}+\text{2}\sqrt{a_{\text{1}}b_{\text{1}}\rho_{\text{1}}})\geq {(a}_{\text{1}}\rho V\text{1}\text{1}-\text{0}.\text{5}\alpha_{\text{1}}+b_{\text{1}}V\text{1}\text{1}+\text{0}.\text{5}\alpha_{\text{1}}+\text{2}\sqrt{a_{\text{1}}b_{\text{1}}V_{\text{1}}}\right)\\ {[l}_{\text{1}}\rho \text{12}+l_{\text{2}}\rho \text{13}+\text{2}\sqrt{a_{\text{1}}b_{\text{1}}}\rho_{\text{1}}]\geq {[l}_{\text{1}}V\text{12}+l_{\text{2}}V\text{13}+\text{2}\sqrt{a_{\text{1}}b_{\text{1}}}V_{\text{1}}] \end{array}$$ (30)

Differentiating (28) yield


$${\dot{V}}_{\text{1}}=-\left|{\dot{q}}_{e\text{0}}\right|=-\frac{\text{1}}{\text{2}}\gamma qevTq_{ev}=\mathrm{\backslash textemdash}\frac{\text{1}}{\text{2}}\gamma \rho_{\text{1}}$$ (31)

In view of (29) and (31), we can obtain that


$${\dot{V}}_{\text{1}}\leq -\frac{\text{1}}{\text{2}}\gamma V_{\text{1}}$$ (32)

If$\left\|q_{ev}\right\|\geq \varepsilon$, by substituting (21) into (31), it follows that


$$\begin{array}{c} {\dot{V}}_{\text{1}}=-\frac{\pi }{\alpha_{\text{1}}T_{\text{1}}\sqrt{a_{\text{1}}b_{\text{1}}}}(a_{\text{1}}\rho_{\text{1}}^{\text{1}-\text{0}.\text{5}\alpha_{\text{1}}}+b_{\text{1}}\rho_{\text{1}}^{\text{1}+\text{0}.\text{5}\alpha_{\text{1}}}+\text{2}\sqrt{a_{\text{1}}b_{\text{1}}}\rho_{1}\text{)}\\ \leq -\frac{\pi }{\alpha_{\text{1}}T_{\text{1}}\sqrt{a_{\text{1}}b_{\text{1}}}}(a_{\text{1}}V_{\text{1}}^{\text{1}-\text{0}.\text{5}\alpha_{\text{1}}}+b_{\text{1}}V_{\text{1}}^{\text{1}+\text{0}.\text{5}\alpha_{\text{1}}}+\text{2}\sqrt{a_{\text{1}}b_{\text{1}}}{\text{V}}_{1}\;) \end{array}$$ (33)

If $\|q_{ev}\;\|<\varepsilon$, it follows that


$$\begin{array}{c} {\dot{V}}_{\text{1}}=-\frac{\pi }{\alpha_{\text{1}}T_{\text{1}}\sqrt{a_{\text{1}}b_{\text{1}}}}(l_{\text{1}}\rho \text{12}+l_{2}\rho \text{13}+\text{2}\sqrt{a_{\text{1}}b_{\text{1}}}\rho_{1}\text{)}\\ \leq -\frac{\pi }{\alpha_{\text{1}}T_{\text{1}}\sqrt{a_{\text{1}}b_{\text{1}}}}(l_{\text{1}}V\text{12}+l_{\text{2}}V\text{13}+\text{2}\sqrt{a_{\text{1}}b_{\text{1}}\;\;}{\text{V}}_{1}\text{)} \end{array}$$ (34)

In view of (33) and (34), it can be concluded that once the sliding surface S=0 is reached, the spacecraft attitude tracking error will converge to $\|q_{ev}\;\|<\varepsilon$ within the predefined time $T_{\text{1}}$.

### 3.2 The design of controller

We develop an ETM as follows


$$\;\;\;\;\;\;\;\;\;\;\;\;\;\;\;\;\;\;\;u(t)=\tilde{u}\left(t_{k}\right)\;\;t\in [t_{k},t_{k+\text{1}})$$ (35)

where $t_{k}$, *k* = 0,1,2... denote the k-th triggering instants. Equation (35) indicates that during $t\in [t_{k},t_{k+\text{1}})$ the control torque u remains equal to $\tilde{u}\left(t_{k}\right)$ which eliminates the need for continuous signal updates and thereby conserves communication resources. In the equations, $\begin{array}{c} \tilde{u}\left(t_{k}\right)\in R^{n} \end{array}$ denotes the continuous virtual controller to be designed, where n represents the dimension of the virtual controller.

The k+1-th triggering instant is defined as


$$\begin{array}{c} t_{k+\text{1}}=\text{inf}\{t>t_{k}|\left|{\tilde{u}}_{\text{1}}(t)-u_{\text{1}}(t)\right|\geq h_{\text{1}}\left|u_{\text{1}}(t)\right|+r_{\text{1}}\;\;\;\text{or}\;\;\;\;\;\;\;\;\;\;\;\;\;\;\;\;\\ \left|{\tilde{u}}_{\text{2}}(t)-u_{\text{2}}(t)\right|\geq h_{\text{2}}\left|u_{\text{2}}(t)\right|+r_{\text{2}}\;\;\;\text{or}\\ \begin{array}{c} \vdots \\ \left|{\tilde{u}}_{n}(t)-u_{n}(t)\right|\geq h_{n}\left|u_{n}(t)\right|+r_{n}\}\;\; \end{array}\; \end{array}$$ (36)

The parameters are event-triggered parameters, 0<h<1 and r>0 are design parameters. According to (35), the relationship between u(t) and $\tilde{u}(t)$ over the interval $t\in [t_{k},t_{k+\text{1}})$ is given by


$$u(t)=\mathrm{\backslash itDelta}\tilde{u}(t)+\upsilon$$ (37)

where $\mathrm{\backslash itDelta}=𝐝𝐢𝐚𝐠[\Delta_{\text{1}},\Delta_{\text{2}},\ldots \Delta_{n}]\in {\mathbb{R}}^{n\times n}$, $\Delta_{i}=\text{1/}[\text{1}+h_{n}{\overset{―}{\lambda }}_{n}(trbrack$, i=1⋯n The unknown time-varying parameters satisfy ${\overset{―}{\lambda }}_{i}(tin[-\text{1},\text{1}]$,which yields $\text{0}<1/(1+h_{max}leq\Delta_{i}\leq \text{1}/(\text{1}-h_{\text{max}})$ with $h_{\text{max}}=\text{max}\left\{h_{\text{1}},h_{\text{2}}..h_{\text{n}}\right\}$, ${\upsilon =[\upsilon_{\text{1}},\upsilon_{\text{2}},\cdots \upsilon_{n}]}^{⊺}\in {\mathbb{R}}^{n}$.where $\upsilon_{n}=\left[r_{n}{\hat{\lambda }}_{n}(t)\right]/[\text{1}+h_{n}{\hat{\lambda }}_{n}(t)]$, and the unknown time – varying parameters ${\hat{\lambda }}_{n}(tin[-\text{1},\text{1}]$,Therefore, ${-r}_{\text{max}}/(\text{1}-h_{\text{max}})\leq \upsilon_{i}\leq r_{\text{max}}/(\text{1}-h_{\text{max}}),r_{\text{max}}=\text{max}\{r_{\text{1}},r_{\text{2}},\cdots r_{\text{n}}\}$, It can be concluded that $\upsilon_{n}$ is bounded.

Remark3

According to the event-triggering mechanism designed in (35) and (36), the controller transmits an updated control input u(t) to the actua*t*or only when the triggering condition (36) is satisfied. Within the interval $t\in [t_{k},t_{k+\text{1}})$, the control input remains constant as $\tilde{u}\left(t_{k}\right)$. Since no communication is required between two consecutive events, the communication load between the controller and the actuator is considerably alleviated during $t\in [t_{k},t_{k+\text{1}})$.

**Fig 2:**
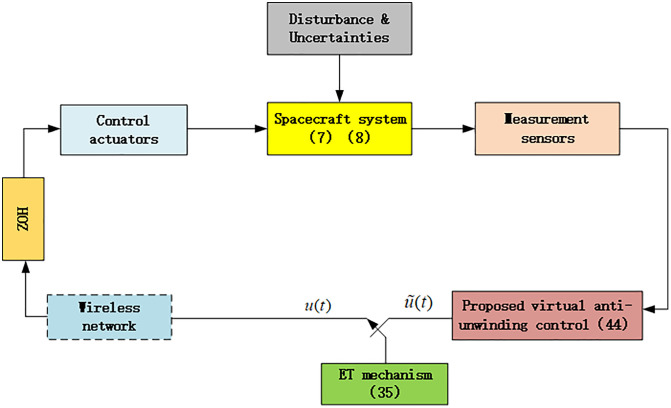
Schematic diagram of the proposed control system.

Fig 2 shows the schematic diagram of the proposed control scheme. It should be noted that the attitude control module can access the sensor measurement information at all times.

Next, combining (6), (7),(18) with (1) leads to


$$\begin{array}{cc} J\dot{S} & =J({\dot{\omega }}_{e}+\text{sign}\left(q_{e\text{0}}\right)\dot{\gamma }q_{ev}+\text{sign}\left(q_{e\text{0}}\right)\gamma {\dot{q}}_{ev}\\ & =-(\omega_{e}+G\omega_{d}{)}^{\times }J_{0}\left(\omega_{e}+G\omega_{d}\right)+J_{\text{0}}\left(\omega_{e}^{\times }G\omega_{d}-G{\dot{\omega }}_{d}\right)+\mathrm{\backslash itDelta}\tilde{u}(t)+\upsilon +d_{\text{1}}\\ & +\left(J_{\text{0}}+\Delta J\right)\text{sign}\left(q_{e\text{0}}\right)\dot{\gamma }q_{ev}+\left(J_{\text{0}}+\Delta J\right)\text{sign}\left(q_{e\text{0}}\right)\gamma {\dot{q}}_{ev}\\ & =-(\omega_{e}+G\omega_{d}{)}^{\times }J_{0}\left(\omega_{e}+G\omega_{d}\right)+J_{\text{0}}\left(\omega_{e}^{\times }G\omega_{d}-G{\dot{\omega }}_{d}\right)+\mathrm{\backslash itDelta}\tilde{u}(t)\\ & {+J}_{0}\text{sign}\left(q_{e\text{0}}\right)\dot{\gamma }q_{ev}+J_{0}\text{sign}\left(q_{e\text{0}}\right)\gamma {\dot{q}}_{ev}+d_{\text{2}}\\ & =F+\mathrm{\backslash itDelta}\tilde{u}(t)+d_{\text{2}} \end{array}$$ (38)

with the residual dynamics $d_{\text{2}}$ and F are defined by


$$\begin{array}{c} d_{\text{2}}=d_{\text{1}}+\upsilon +\Delta J\text{sign}\left(q_{e\text{0}}\right)\dot{\gamma }q_{ev}+\Delta J\text{sign}\left(q_{e\text{0}}\right)\gamma {\dot{q}}_{ev}=-(\omega_{e}+G\omega_{d}{)}^{\times }\Delta J\left(\omega_{e}+G\omega_{d}\right)+d+\upsilon +\Delta J(\text{sign}\left(q_{e\text{0}}\right)\dot{\gamma }q_{ev}+\text{sign}\left(q_{e\text{0}}\right)\gamma {\dot{q}}_{ev}+\left(\omega_{e}^{\times }G\omega_{d}-G{\dot{\omega }}_{d}\right)) \end{array}$$ (39)

$$\begin{array}{c} F=-(\omega_{e}+G\omega_{d}{)}^{\times }J_{0}\left(\omega_{e}+G\omega_{d}\right)+J_{0}\left(\omega_{e}^{\times }G\omega_{d}-G{\dot{\omega }}_{d}\right)\\ +J_{0}\text{sign}\left(q_{e\text{0}}\right)\dot{\gamma }q_{ev}+J_{0}\text{sign}\left(q_{e\text{0}}\right)\gamma {\dot{q}}_{ev} \end{array}$$ (40)

To ensure the anti-unwinding performance outside the sliding mode surface, the Lyapunov function is designed as follows


$$V_{\text{2}}=\text{0}.\text{5}{q_{e\text{0}}}^{-\text{2}}S^{T}JS$$ (41)

**Remark 4.** This section aims to describe the properties of the proposed function. On the sliding surface S=0, the convergence of the spacecraft’s attitude error and its anti-unwinding behavior can be guaranteed. A controller will then be designed to ensure that the sliding surface converges to S=0 as possible, while maintaining anti-unwinding behavior even outside the sliding surface. Therefore, as long as the Lyapunov function remains bounded, the anti-unwinding property of the spacecraft is ensured; and as long as the Lyapunov function converges to zero, convergence of the sliding surface to zero is also guaranteed.

The above statements hold under the assumption of no external disturbances and no inertia uncertainties. However, in the presence of inertia uncertainties and external disturbances, we can only prove that the sliding surface converges to a bounded region within a predefined time (as ensured by the controller). Based on this, it can then be shown that the attitude error and angular velocity error also converge to bounded regions, which are determined by the boundedness of the sliding surface and the properties of its design.

Taking the derivative of $V_{\text{2}}$ along (41), we have


$$\begin{array}{cc} {\dot{V}}_{\text{2}} & =-q_{e\text{0}}^{-\text{3}}{\dot{q}}_{e\text{0}}S^{⊺}JS+\text{0}.\text{5}{q_{e\text{0}}}^{-\text{2}}S^{⊺}\Delta \dot{J}S+{q_{e\text{0}}}^{-\text{2}}S^{⊺}J\dot{S}\\ \left[4pt\right] & =S^{⊺}\left[-q_{e\text{0}}^{-\text{3}}{\dot{q}}_{e\text{0}}JS+\text{0}.\text{5}{q_{e\text{0}}}^{-\text{2}}\Delta \dot{J}S+{q_{e\text{0}}}^{-\text{2}}J\dot{S}\right]\\ \left[4pt\right] & {=S}^{⊺}[-q_{e\text{0}}^{-\text{3}}{\dot{q}}_{e\text{0}}JS+\text{0}.\text{5}{q_{e\text{0}}}^{-\text{2}}\Delta \dot{J}S+{q_{e\text{0}}}^{-\text{2}}F+{q_{e\text{0}}}^{-\text{2}}\mathrm{\backslash itDelta}\tilde{u}(t)+{q_{e\text{0}}}^{-\text{2}}d_{\text{2}}]\\ \left[4pt\right] & {=S}^{⊺}\left[{q_{e\text{0}}}^{-\text{2}}F+{q_{e\text{0}}}^{-\text{2}}\mathrm{\backslash itDelta}\tilde{u}(t)+d_{\text{3}}\right]\\ \left[4pt\right] & {=S}^{⊺}\left[F_{\text{1}}+{q_{e\text{0}}}^{-\text{2}}\mathrm{\backslash itDelta}\tilde{u}(t)+d_{\text{3}}\right] \end{array}$$ (42)

where $F_{\text{1}}{=q_{e\text{0}}}^{-\text{2}}F$is known and $d_{\text{3}}=q_{e\text{0}}^{-\text{2}}d_{\text{2}}-q_{e\text{0}}^{-\text{3}}{\dot{q}}_{e\text{0}}JS+\text{0}.\text{5}q_{e\text{0}}^{-\text{2}}\Delta \dot{J}S$ is unknown.

Combining **assumptions 1, 2** and (39) yields


$$d_{\text{3}}\leq b^{⊺}v$$ (43)

where $v={\left[\left(\left\|q_{e\text{0}}^{-\text{3}}{\dot{q}}_{e\text{0}}S\right\|+q_{e\text{0}}^{-\text{2}}\left\|\omega \right\|\right),\left\|\text{0}.\text{5}q_{e\text{0}}^{-\text{2}}S\right\|,q_{e\text{0}}^{-\text{2}}\left\|\lambda \right\|,q_{e\text{0}}^{-\text{2}}\right]}^{⊺}$, $\lambda =\text{sign}\left(q_{e\text{0}}\right)\dot{\gamma }q_{ev}+\text{sign}\left(q_{e\text{0}}\right)\gamma {\dot{q}}_{ev}+\left(\omega_{e}^{\times }G\omega_{d}-G{\dot{\omega }}_{d}\right)$. $b\in {\mathbb{R}}^{\text{4}}$, where all elements of b are bounded. $\hat{b}$ is the estimate of b, and $\tilde{b}=\hat{b}-b$ is the estimation error.

**Remark 5.** It can be seen that through the intermediate transformation involving $d_{\text{1}}$ and $d_{\text{2}}$, all model uncertainties are eventually aggregated into $d_{\text{3}}$. This results in a formulation involving the known term $F_{\text{1}}$, the unknown term $d_{\text{3}}$, and the controller to be designed. The term $d_{\text{3}}$ encapsulates both unknown disturbances and parametric uncertainties. Therefore, it has been shown that $d_{\text{3}}$ converges to a bounded region.

An event-triggered adaptive predefined-time anti-unwinding controller is proposed.


$$\tilde{u}=-\frac{\left(\text{1}+h^{\prime }\right)}{V_{r}}\left(\frac{F^{⊤}F}{\sqrt{F^{⊤}FS^{⊤}S+\epsilon_{\text{1}}^{\text{2}}}}+\frac{\chi^{⊤}\chi }{\sqrt{\chi^{⊤}\chi S^{⊤}S+\epsilon_{\text{1}}^{\text{2}}}}+\frac{d_{r}^{\text{2}}}{\sqrt{d_{r}^{\text{2}}S^{⊤}S+\epsilon_{\text{1}}^{\text{2}}}}+V_{r}\Omega_{\text{1}}\right)S$$ (44)

The adaptive update law of $\hat{b}$ is designed as


$$\dot{\hat{b}}=\sigma \left(\left\|S\right\|v-\hat{b}\right)$$ (45)

where $d_{r}={\hat{b}}^{⊤}v,V_{r}=q_{e\text{0}}-\text{2},h^{\prime }=h_{\text{max}}=\text{max}\{h_{\text{1}},h_{\text{2}},\ldots h_{n}\},\Omega_{\text{1}}>\text{0}$,$\epsilon_{\text{1}}>\text{0},\sigma >\text{0}$

where$\chi =\frac{\pi }{\alpha_{\text{2}}T_{\text{2}}\sqrt{a_{\text{2}}b_{\text{2}}}}\left(a_{\text{2}}V_{r}^{\text{1}-\text{0}.\text{5}\alpha_{\text{2}}}{\text{sig}}^{\text{1}-\alpha_{\text{2}}}\left(S\right)+b_{\text{2}}V_{r}^{\text{1}+\text{0}.\text{5}\alpha_{\text{2}}}{\text{sig}}^{\text{1}+\alpha_{\text{2}}}\left(S\right)+\text{2}\sqrt{a_{\text{2}}b_{\text{2}}}V_{r}S\right)$ and $\alpha_{\text{2}},a_{\text{2}},T_{\text{2}},b_{\text{2}}$are adjustable parameters defined by equation (44).where$\text{0}<a_{\text{2}}<\text{1},b_{\text{2}}>\text{0},a_{\text{2}}>\text{0}$,$T_{\text{2}}>\text{0}$.

**Theorem 2.** For the spacecraft attitude error systems (7), (8), the designed predefined-time sliding surface (20), the proposed controller (44), adaptive law (45), and event-triggering mechanism (35), under the given conditions, ensure that the spacecraft achieves the following conclusions

(1) Inside and outside the sliding surface, the system consistently exhibits anti-unwinding behavior; all signals in the closed-loop system are bounded.
(2) The sliding surface S, spacecraft attitude error $q_{ev}$, and angular velocity error$\;\omega_{e}$ will converge to a neighborhood around zero within the predefined time.
(3) There exists a minimum positive constant $\overset{―}{T}$ such that the triggering interval at any two adjacent time instants satisfies $T_{k}\geq \overset{―}{T}$. Therefore, the designed control strategy can avoid the Zeno phenomenon.

**Proof (1).** Select the Lyapunov function as follows


$$\begin{array}{c} V_{\text{3}}=V_{\text{2}}+\frac{{\tilde{b}}^{⊤}\tilde{b}}{2\sigma }\;\;\;\;\;\;\;\;\;\\ =\text{0}.\text{5}q_{e\text{0}}-\text{2}S^{⊤}JS+\frac{{\tilde{b}}^{⊤}\tilde{b}}{2\sigma } \end{array}$$ (46)

Calculating the time derivative (46) produces


$$\begin{array}{c} {\dot{V}}_{\text{3}}=\text{0}.\text{5}q_{e\text{0}}-\text{2}S^{⊤}JS+\frac{{\tilde{b}}^{⊤}\dot{\hat{b}}}{\sigma }\;\;\;\\ =S^{⊤}\left[F_{\text{1}}+q_{e\text{0}}-\text{2}\Delta \tilde{u}(t)+d_{\text{3}}\right] \end{array}$$ (47)

Combining **Lemma 1**, **Lemma 2,** and (44) yields


$$\begin{array}{c} S^{⊤}q_{e\text{0}}-\text{2}\Delta \tilde{u}(t)\leq -\left(\frac{F^{⊤}F}{\sqrt{F^{⊤}FS^{⊤}S+\epsilon_{\text{1}}^{\text{2}}}}+\frac{\chi^{⊤}\chi }{\sqrt{\chi^{⊤}\chi S^{⊤}S+\epsilon_{\text{1}}^{\text{2}}}}+\frac{d_{r}^{\text{2}}}{\sqrt{d_{r}^{\text{2}}S^{⊤}S+\epsilon_{\text{1}}^{\text{2}}}}+V_{r}\Omega_{\text{1}}\right)S^{⊤}S\\ \leq \text{3}\epsilon_{\text{1}}-\left(\left\|F\right\|\left\|S\right\|+\chi^{⊤}S+\left\|{\hat{b}}^{⊤}v\right\|\left\|S\right\|\right)-V_{r}\Omega_{\text{1}}S^{⊤}S \end{array}$$ (48)

Substituting (48) into (47) leads to


$$\begin{array}{c} {\dot{V}}_{\text{3}}\leq \left\|S\right\|\left\|F\right\|+\text{3}\epsilon_{\text{1}}-\left(\left\|F\right\|\left\|S\right\|+\chi^{⊤}S+\left\|{\hat{b}}^{⊤}v\right\|\left\|S\right\|\right)-V_{r}\Omega_{\text{1}}S^{⊤}S+\left\|S\right\|\left(b^{⊤}v\right)+\frac{{\tilde{b}}^{⊤}\dot{\hat{b}}}{\sigma }\\ \leq \text{3}\epsilon_{\text{1}}-\chi^{⊤}S-\left({\hat{b}}^{⊤}v\right)\left\|S\right\|-V_{r}\Omega_{\text{1}}S^{⊤}S+\left(b^{⊤}v\right)\left\|S\right\|+\frac{{\tilde{b}}^{⊤}\dot{\hat{b}}}{\sigma }\\ \leq -\chi^{⊤}S-V_{r}\Omega_{\text{1}}S^{⊤}S-{\tilde{b}}^{⊤}v\left\|S\right\|+\text{3}\epsilon_{\text{1}}+\frac{{\tilde{b}}^{⊤}\dot{\hat{b}}}{\sigma }\\ \leq -\chi^{⊤}S-V_{r}\Omega_{\text{1}}S^{⊤}S+\frac{\text{1}}{\text{2}}{\tilde{b}}^{⊤}\hat{b}+\text{3}\epsilon_{\text{1}}\\ \leq -V_{r}\Omega_{\text{1}}S^{⊤}S-\frac{\text{1}}{\text{2}}{\tilde{b}}^{⊤}\tilde{b}+\frac{\text{1}}{\text{2}}b^{⊤}b+\text{3}\epsilon_{\text{1}}\\ \leq -\delta V_{\text{3}}+\phi \end{array}$$ (49)

where$\delta =\text{min}\{\frac{\text{1}}{\lambda_{\text{max}}(J)},\sigma \}>\text{0}$, $\begin{array}{c} \text{0}<\phi =\frac{\text{1}}{\text{2}}b^{T}b+\text{3}\epsilon_{\text{1}}<\infty \end{array}$ is bounded.

**Remark 6.** The inertia matrix J is symmetric and positive definite, and the following inequality is satisfied [38]


$$\lambda_{\text{min}}\left(J\right){\left\|x\right\|}^{\text{2}}\leq x^{T}Jx\leq \lambda_{\text{max}}\left(J\right){\left\|x\right\|}^{\text{2}},\forall J\in R^{\text{3}}$$ (50)

where $\lambda_{\text{min}}\left(J\right)$and $\lambda_{\text{max}}\left(J\right)$ are the minimum and maximum eigenvalues of Jrespectively.

Combining (46) and (49), it follows that $\;V_{\text{3}}$ is bounded. Consequently,$V_{\text{2}}$ is bounded and the estimation error $\tilde{b}$. However, since $V_{\text{2}}=\text{0}.\text{5}q_{e\text{0}}-\text{2}S^{T}JS$, the following three scenarios exist and need to be analyzed individually

(1) $q_{e\text{0}}-\text{2}\to \infty ,S\to \text{0}$

According to the definition of the sliding surface (20), $S\to \text{0}⟺\omega_{e}\Rightarrow -\text{sign}(q_{e\text{0}})\gamma q_{ev}$. However, when $\omega_{e}=-\text{sign}(q_{e\text{0}})\gamma q_{ev}$, according to **Theorem 1**, the spacecraft necessarily exhibits anti-unwinding behavior, and $q_{e\text{0}}-\text{2}\to \infty$ cannot occur. This scenario is contradictory.

(2) $q_{e\text{0}}-\text{2}\to \text{0},S\to \infty$

According to the property of $q_{e\text{0}}$, $\text{1}\leq q_{e\text{0}}-\text{2}\leq \infty$.Therefore, this scenario cannot be satisfied.

(3) $q_{e\text{0}}-\text{2}$ is bounded, S is bounded

Based on the above analysis, only the third scenario is valid. Therefore, as long as $V_{\text{2}}$ is bounded, then the sliding surface S is bounded, and the potential function $V_{r}=q_{e\text{0}}^{-\text{2}}$ is inevitably bounded. This ensures that the spacecraft inevitably exhibits anti-unwinding behavior.

Based on the foregoing analysis, throughout the spacecraft’s attitude tracking control process, there necessarily exist positive constants $m_{\text{1}},m_{\text{2}},m_{\text{3}},m_{\text{4}},m_{\text{5}}>\text{0}$,satisfying $\left\|S\right\|\leq m_{\text{1}},V_{r}\leq m_{\text{2}},\left\|\chi \right\|\leq m_{\text{3}},\left\|\tilde{b}\right\|\leq m_{\text{4}},\left\|v\right\|\leq m_{\text{5}}$.

Furthermore, due to the properties of the attitude quaternion, the attitude error is bounded, and consequently, $\text{sign}(q_{e\text{0}})\gamma q_{ev}$ is bounded. Since the sliding surface S is bounded and considering its definition (20), the angular velocity error $\omega_{e}$ is bounded. Additionally, since the estimation error $\tilde{b}$ is bounded and the true value b is bounded, the estimated value $\hat{b}$. Therefore, the virtual controller $\tilde{u}$ is bounded. This completes the proof of statement (1).

**Proof (2)** To prove the convergence of the sliding surface S, attitude error $q_{ev}$, and angular velocity error $\omega_{e}$, we reformulate equation (42) as follows


$$\begin{array}{cc} \dot{V}\text{2}= & ST[F\text{1}+qe\text{0}-\text{2}\Delta \tilde{u}(t)+d\text{3}\backslash \\ & \leq \left\|S\right\|\left\|F\right\|+\left\|S\right\|\left(bTv\right)+\text{3}\epsilon \text{1}-\left(\left\|F\right\|\left\|S\right\|+\chi TS+\left\|\hat{b}Tv\right\|\left\|S\right\|\right)-Vr\Omega \text{1}STS\\ & \leq -\chi TS-Vr\Omega \text{1}STS-\tilde{b}Tv\left\|S\right\|+\text{3}\epsilon \text{1}\\ & \leq -\chi TS+\phi \text{1} \end{array}$$ (51)

where $\varphi_{\text{1}}=\text{3}\epsilon_{\text{1}}+m_{\text{1}}m_{\text{4}}m_{\text{5}}$ is bounded, satisfy $\text{0}<\phi_{\text{1}}<\infty$.

In light of **Lemmas 4** and χ, we obtain


$$\begin{array}{c} -\chi^{⊤}S=\frac{\pi }{\alpha_{\text{2}}T_{\text{2}}\sqrt{a_{\text{2}}b_{\text{2}}}}\left(a_{\text{2}}V_{r}^{\text{1}-\text{0}.\text{5}\alpha_{\text{2}}}\sum_{i=\text{1}}^{\text{3}}{\left|s_{i}\right|}^{\text{2}-\alpha_{\text{2}}}+b_{\text{2}}V_{r}^{\text{1}+\text{0}.\text{5}\alpha_{\text{2}}}\sum_{i=\text{1}}^{\text{3}}{\left|s_{i}\right|}^{\text{2}+\alpha_{\text{2}}}+\text{2}\sqrt{a_{\text{2}}b_{\text{2}}}V_{r}S^{⊤}S\right)\\ \leq -\frac{\pi }{\alpha_{\text{2}}T_{\text{2}}\sqrt{a_{\text{2}}b_{\text{2}}}}\left(a_{\text{2}}V_{r}^{\text{1}-\text{0}.\text{5}\alpha_{\text{2}}}{\sum_{i=\text{1}}^{\text{3}}\left(s_{i}^{\text{2}}\right)}^{\text{1}-\text{0}.\text{5}\alpha_{\text{2}}}+b_{\text{2}}V_{r}^{\text{1}+\text{0}.\text{5}\alpha_{\text{2}}}{\sum_{i=\text{1}}^{\text{3}}\left(s_{i}^{\text{2}}\right)}^{\text{1}+\text{0}.\text{5}\alpha_{\text{2}}}+\frac{\text{4}}{\lambda_{\text{max}}\left(J\right)}\sqrt{a_{\text{2}}b_{\text{2}}}V_{\text{2}}\right)\\ \leq -\frac{\pi }{\alpha_{\text{2}}T_{\text{2}}\sqrt{a_{\text{2}}b_{\text{2}}}}\left(a_{\text{2}}V_{r}^{\text{1}-\text{0}.\text{5}\alpha_{\text{2}}}{\left[\sum_{i=\text{1}}^{\text{3}}\left(s_{\text{1}}^{\text{2}}\right)\right]}^{\text{1}-\text{0}.\text{5}\alpha_{\text{2}}}+{\text{3}}^{-\frac{\alpha_{\text{2}}}{\text{2}}}b_{\text{2}}V_{r}^{\text{1}+\text{0}.\text{5}\alpha_{\text{2}}}{\left[\sum_{i=\text{1}}^{\text{3}}\left(s_{\text{1}}^{\text{2}}\right)\right]}^{\text{1}+\text{0}.\text{5}\alpha_{\text{2}}}+\frac{\text{4}}{\lambda_{\text{max}}(J)}\sqrt{a_{\text{2}}b_{\text{2}}}V_{\text{2}}\right)\\ \leq -\frac{\pi }{\alpha_{\text{2}}T_{\text{2}}\sqrt{a_{\text{2}}b_{\text{2}}}}\left(a_{\text{2}}{\left(V_{r}S^{⊤}S\right)}^{\text{1}-\text{0}.\text{5}\alpha_{\text{2}}}+{\text{3}}^{-\frac{\alpha_{\text{2}}}{\text{2}}}b_{\text{2}}{\left(V_{r}S^{⊤}S\right)}^{\text{1}+\text{0}.\text{5}\alpha_{\text{2}}}+\frac{\text{4}}{\lambda_{\text{max}}\left(J\right)}\sqrt{a_{\text{2}}b_{\text{2}}}V_{\text{2}}\right)\\ \leq -\frac{\pi }{\alpha_{\text{2}}T_{\text{2}}\sqrt{a_{\text{2}}b_{\text{2}}}}\left(a_{\text{2}}{\left(\frac{\text{2}}{\lambda_{\text{max}}\left(J\right)}V_{\text{2}}\right)}^{\text{1}-\text{0}.\text{5}\alpha_{\text{2}}}+{\text{3}}^{-\frac{\alpha_{\text{2}}}{\text{2}}}b_{\text{2}}{\left(\frac{\text{2}}{\lambda_{\text{max}}\left(J\right)}V_{\text{2}}\right)}^{\text{1}+\text{0}.\text{5}\alpha_{\text{2}}}+\frac{\text{4}}{\lambda_{\text{max}}\left(J\right)}\sqrt{a_{\text{2}}b_{\text{2}}}V_{\text{2}}\right)\\ \leq -\frac{\pi }{\alpha_{\text{2}}T_{\text{21}}\sqrt{a_{\text{2}}b_{\text{2}}}}\left(a_{\text{2}}{\left(V_{\text{2}}\right)}^{\text{1}-\text{0}.\text{5}\alpha_{\text{2}}}+b_{\text{2}}{\left(V_{\text{2}}\right)}^{\text{1}+\text{0}.\text{5}\alpha_{\text{2}}}+\text{2}\sqrt{a_{\text{2}}b_{\text{2}}}V_{\text{2}}\right) \end{array}$$ (52)

where $T_{\text{21}}=T_{\text{2}}/\beta$,$\beta =\text{max}\left\{{\left[\text{2}/\lambda_{\text{max}}(J)\right]}^{\text{0}.\text{5}\alpha_{\text{2}}-\text{1}},{\text{3}}^{\frac{\alpha_{\text{2}}}{\text{2}}}{\left[\text{2}/\lambda_{\text{max}}(J)\right]}^{\text{0}.\text{5}\alpha_{\text{2}}+\text{1}},\text{2}/\lambda_{\text{max}}(J)\right\}$.

Combining (51) and (52),we can


$$\begin{array}{c} {\dot{V}}_{\text{2}}\leq -\chi^{⊤}S+\phi_{\text{1}}\\ \leq -\frac{\pi }{\alpha_{\text{2}}T_{\text{21}}\sqrt{a_{\text{2}}b_{\text{2}}}}\left(a_{\text{2}}{\left(V_{\text{2}}\right)}^{\text{1}-\text{0}.\text{5}\alpha_{\text{2}}}+b_{\text{2}}{\left(V_{\text{2}}\right)}^{\text{1}+\text{0}.\text{5}\alpha_{\text{2}}}+\text{2}\sqrt{a_{\text{2}}b_{\text{2}}}V_{\text{2}}\right)+\phi_{\text{1}} \end{array}$$ (53)

In light of **Lemmas 3**, $V_{\text{2}}$ will converge to the following residual set within the predefined time $T_{\text{2}\text{2}}=\sqrt{\text{2}}T_{\text{2}\text{1}}$.


$$V_{\text{min}\text{2}}=\left\{\underset{t\to T_{\text{2}\text{2}}}{\text{lim}}x|V_{\text{2}}(x)\leq \text{min}\left\{{\left(\frac{\text{2}\phi_{\text{1}}\alpha_{\text{2}}\sqrt{a_{\text{2}}b_{\text{2}}}}{a_{\text{2}}\pi }\right)}^{\frac{\text{2}}{\text{2}-\alpha_{\text{2}}}},{\left(\frac{\text{2}\phi_{\text{1}}\alpha_{\text{2}}\sqrt{a_{\text{2}}b_{\text{2}}}}{b_{\text{2}}\pi }\right)}^{\frac{\text{2}}{\text{2}+\alpha_{\text{2}}}}\right\}\right\}$$ (54)

Since $V_{r}\leq m_{\text{2}}$, we can obtain from (38) that


$$\left\|S\right\|\leq \frac{\text{2}V_{\text{min}\text{2}}}{m_{\text{2}}\lambda_{\text{min}}(J)}$$ (55)

Since the sliding surface employs a partitioned design, the convergence of attitude error and angular velocity error must be analyzed separately for each region. The following analysis addresses the case where $q_{e\text{0}}(t_{s})>\text{0}$. The case for follows an analogous structure and is not explicitly discussed here.

If $\left\|q_{ev}\right\|\geq \varepsilon$, combining (20), (31) can be rewritten as


$${\dot{V}}_{\text{1}}=-\left|{\dot{q}}_{e\text{0}}\right|=-\frac{\text{1}}{\text{2}}\gamma q_{ev}^{⊤}q_{ev}+\frac{\text{1}}{\text{2}}q_{ev}^{⊤}S$$ (56)

Combining (33),we can obtain


$${\dot{V}}_{\text{1}}\leq -\frac{\pi }{\alpha_{\text{1}}T_{\text{1}}\sqrt{a_{\text{1}}b_{\text{1}}}}\left(a_{\text{1}}V_{\text{1}}^{\text{1}-\text{0}.\text{5}\alpha_{\text{1}}}+b_{\text{1}}V_{\text{1}}^{\text{1}+\text{0}.\text{5}\alpha_{\text{1}}}+\text{2}\sqrt{a_{\text{1}}b_{\text{1}}}V_{\text{1}}\right)+\text{0}.\text{5}m_{\text{1}}$$ (57)

In light of the **Lemmas 3**, $V_{\text{1}}$ will converge to the following residual set within the predefined time $\sqrt{2}T_{1}+\sqrt{2}T_{21}$.


$$V_{\text{min}\text{1}}=\left\{\underset{t\to \sqrt{\text{2}}T_{\text{1}}}{\text{lim}}x|V_{\text{1}}(x)\leq \text{min}\left\{{\left(\frac{m_{\text{1}}\alpha_{\text{1}}\sqrt{a_{\text{1}}b_{\text{1}}}}{a_{\text{1}}\pi }\right)}^{\frac{\text{2}}{\text{2}-\alpha_{\text{1}}}},{\left(\frac{m_{\text{1}}\alpha_{\text{1}}\sqrt{a_{\text{1}}b_{\text{1}}}}{b_{\text{1}}\pi }\right)}^{\frac{\text{2}}{\text{2}+\alpha_{\text{1}}}}\right\}\right\}$$ (58)

Based on the analysis, $\text{1}-\left|q_{e\text{0}}\right|$ converges to a neighborhood around 0,implying that $\left|q_{e\text{0}}\right|$ converges to a neighborhood around 1, within the convergence domain $\text{1}\geq \left|q_{e\text{0}}\right|\geq \eta ,\text{0}<\eta \leq \text{1}$. Given the quaternion normalization constraint $q_{ev}^{T}q_{ev}+q_{e\text{0}}^{\text{2}}=\text{1}$., the attitude error satisfies $\left\|q_{ev}\right\|\leq \sqrt{\text{1}-\eta^{\text{2}}}$. Consequently, within the predefined time $\sqrt{2}T_{1}+\sqrt{2}T_{21}$, the attitude error converges to


$$\left\|q_{ev}\right\|\leq \text{max}\{\sqrt{\text{1}-\eta^{\text{2}}},\sqrt{\varepsilon }\}=\varepsilon_{q}$$ (59)

Combining the definition of the sliding surface (20), it follows that within the predefined time $\sqrt{\text{2}}T_{\text{1}}+\sqrt{\text{2}}T_{\text{2}\text{1}}$


$$\left\|\omega_{e}\right\|\leq m_{\text{1}}+\frac{\text{2}\pi }{\alpha_{\text{1}}T_{\text{1}}\sqrt{a_{\text{1}}b_{\text{1}}}}\left(a_{\text{1}}\epsilon_{q}^{\alpha_{\text{1}}}+b_{\text{1}}\epsilon_{q}^{\alpha_{\text{1}}}+\text{2}\sqrt{a_{\text{1}}b_{\text{1}}}\epsilon_{q}\right)$$ (60)

In summary, since the sliding surface S and attitude error $q_{ev}$ converge, and the sliding surface definition ensures the convergence of the angular velocity error $\omega_{e}$, **the proof of statement (2)** is complete.

**Proof (3).** A contradiction-based proof is constructed to rigorously demonstrate the exclusion of Zeno phenomena in the proposed event-triggered mechanism. Suppose there exist two consecutive triggering instants with a diminishing time interval$T_{k}=t_{k+\text{1}}-t_{k}\to \text{0}$

It can be derived that


$$\underset{T_{k}\to \text{0}}{\text{lim}}\left|{\tilde{u}}_{i}\left(t_{k}+T_{k}\right)-{\tilde{u}}_{i}\left(t_{k}\right)\right|=\text{0}$$ (61)

However, the event-triggering policy inherently requires that for any $t\in [t_{k},t_{k+\text{1}})$.


$$\left|{\tilde{u}}_{i}\left(t_{k}+T_{k}\right)-{\tilde{u}}_{i}\left(t_{k}\right)\right|>h_{i}\left|{\tilde{u}}_{l_{\text{1}}}\left(t_{k}\right)\right|+r_{i}>\text{0}$$ (62)

The mutual exclusivity between (61) and (62) invalidates the initial assumption. Consequently, there must exist a strictly positive constant $\overset{―}{T}$, such that all adjacent triggering intervals satisfy $T_{k}\geq \overset{―}{T}$, This conclusively proves that the designed control strategy theoretically precludes Zeno behavior.

## 4. Simulation results

To validate the effectiveness of the designed controller (44), this section conducts numerical simulations comparing it with the finite-time anti-unwinding controller from [17] and the fixed-time anti-unwinding controller in [21].

In the section of the predefined-time sliding mode surface and the controller, the corresponding parameter ranges have been provided, and we select the parameters within these ranges.

Selecting appropriate parameters is necessary for controller application. To achieve smaller stability error, ε are required to be smaller. For the predefined-time sliding mode surface, the parameters $T_{\text{1}}=\text{20}$ and $T_{\text{2}}=\text{20}$ were selected to ensure that the system stabilizes within a reasonable time while accounting for the system’s dynamic response. To balance convergence time, the parameters $\alpha_{\text{1}}=\text{0}.\text{6}$ and $a_{\text{1}}=\text{0}.\text{6}$.The boundary layer parameters $b_{\text{1}}=\text{1},b_{\text{2}}=\text{1}$ were chosen to reduce chattering near the sliding surface, ensuring smooth operation of the system without introducing unnecessary oscillations. The parameter r and h play a critical role in the event-triggered mechanism, specifically in adjusting the triggering threshold. we selected h=0.8,r=0.01. These values were chosen to ensure that control updates were sent frequently enough to maintain system performance, without unnecessarily overloading the communication system.

The parameters of the proposed controller, initial conditions of the spacecraft dynamics, and relevant coefficients are provided in Table 1.

**Table 1:** Simulation parameters.

Indexe	Item	Value
Design Parame-ters	$T_{\text{1}},T_{\text{2}}$	20,20
	$b_{\text{1}},b_{\text{2}},\varepsilon$	1,1,${\text{10}}^{-\text{7}}$
	$h_{\text{1}},h_{\text{2}},h_{\text{3}},h_{\text{4}}$	0.8,0.8,0.8,0.8
	$r_{\text{1}},r_{\text{2}},r_{\text{3}},r_{\text{4}}$	0.01,0.01,0.01,0.01
	$\epsilon_{\text{1}},\Omega_{\text{1}},\sigma$	0.1,15,0.1
	$\alpha_{\text{1}},a_{\text{1}},\alpha_{\text{2}},a_{\text{2}}$	0.6,1,0.6,1
Initial states	$\omega_{\text{0}}$	[−0.1;−0.1;0.1]
	$q_{\text{0}}$	[0.8074;−0.539;0;0.240]
	$\hat{b}(\text{0})$	[0.1;0.1;0.1;0.1]
	$J_{\text{0}}$	diag(10,12,14)
	ΔJ	diag(30[0.15sin(0.2t),0.25cos(0.4t),0.20sin(0.3t)])
	d	0.02[sin(0.4t)+1;cos(0.4t)+1;sin(0.4t)+1]
	$q_{d}$	[−0.8043;−0.3;−0.3;0.3375]
	$\omega_{d}$	0.05[sin(0.01πt);sin(0.02πt);sin(0.03πt)]

**Fig 3:**
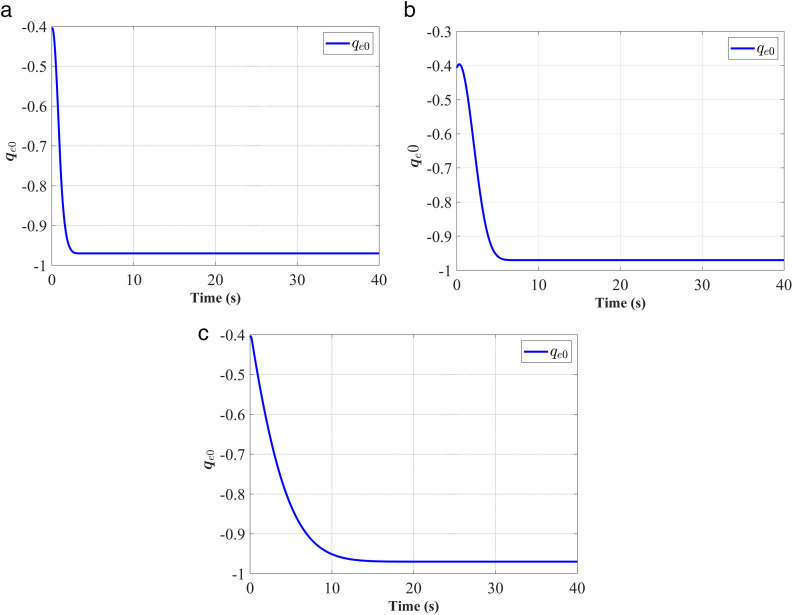
Response of the attitude quaternion vector part ${𝐪}_{e0}$. (a) represent the proposed controller; (b) represent the fixed-time anti-unwinding controller from [21]. (c) represent the finite-time anti-unwinding controller from [17].

**Fig 4:**
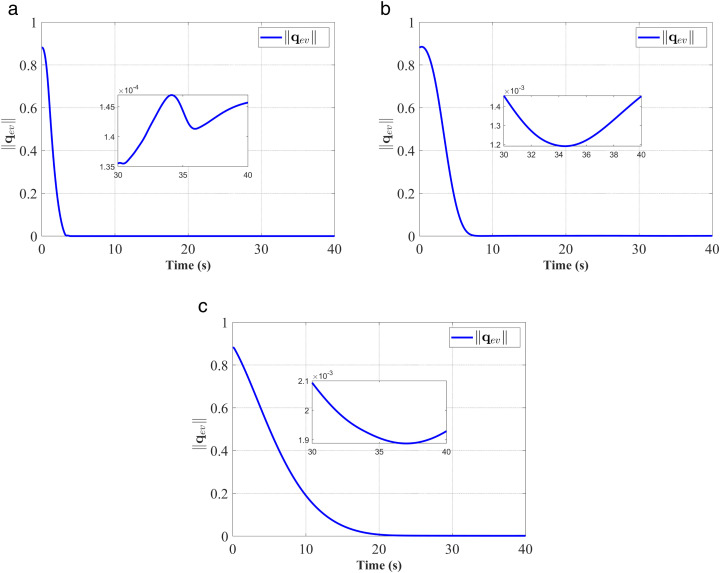
Response of $\left\|{𝐪}_{e}\right\|$. (a) represent the proposed controller; (b) represent the fixed-time anti-unwinding controller from [21]. (c) represent the finite-time anti-unwinding controller from [17].

**Fig 5:**
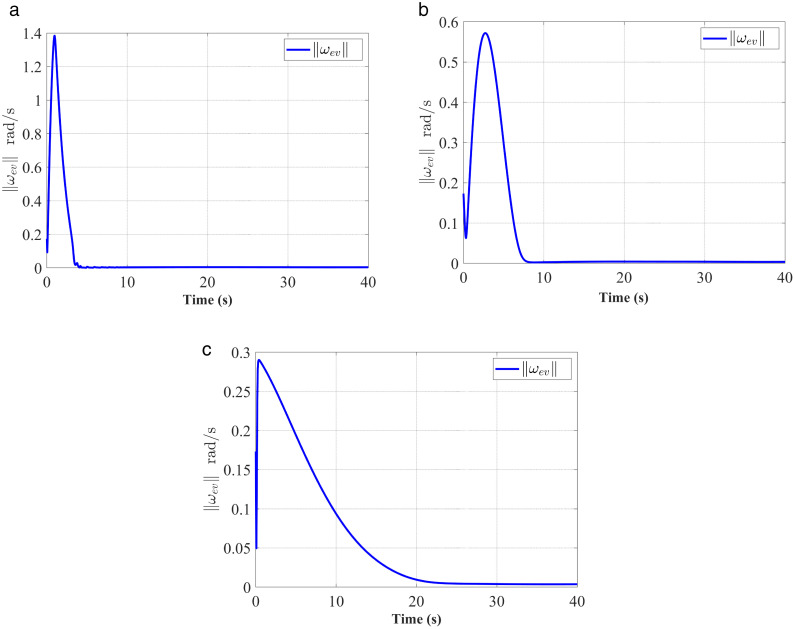
Response of $\left\|\omega_{ev}\right\|$. (a) represent the proposed controller; (b) represent the fixed-time anti-unwinding controller from [21]. (c) represent the finite-time anti-unwinding controller from [17].

**Fig 6:**
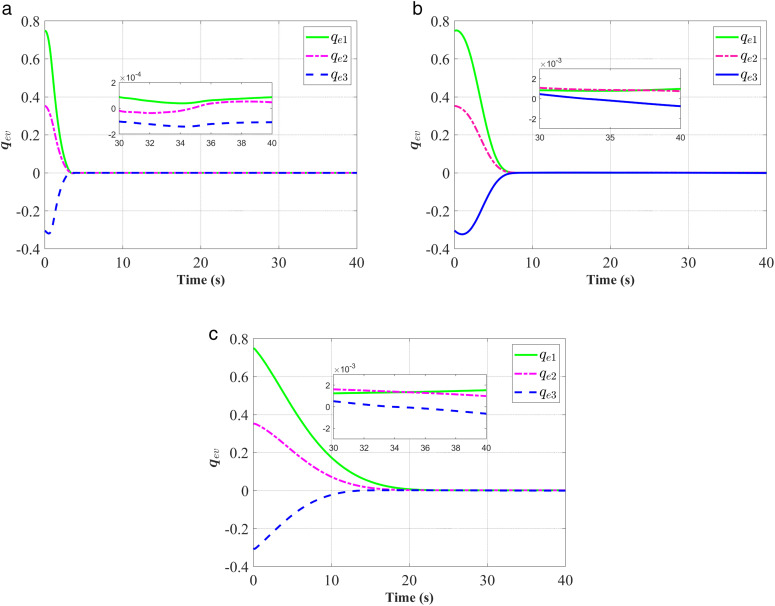
Response of the attitude error${𝐪}_{ev}$. (a) represent the proposed controller; (b) represent the fixed-time anti-unwinding controller from [21]. (c) represent the finite-time anti-unwinding controller from [17].

**Fig 7:**
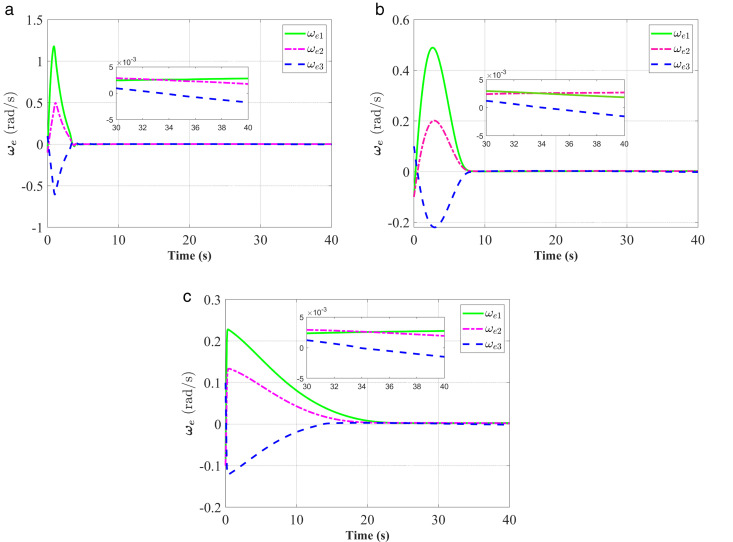
Response of the angular velocity error $\omega_{e}$. (a) represent the proposed controller; (b) represent the fixed-time anti-unwinding controller from [21]. (c) represent the finite-time an-ti-unwinding controller from [17].

**Fig 8:**
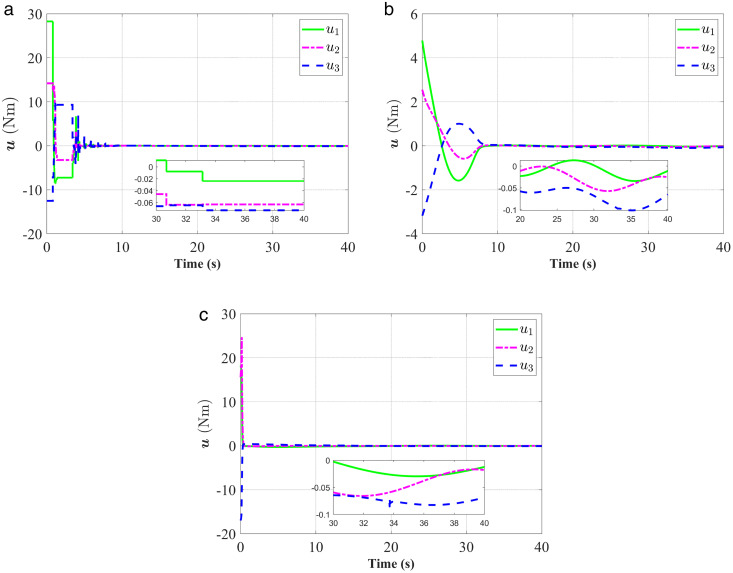
Response of the control torque 𝐮. (a) represent the proposed controller; (b) represent the fixed-time anti-unwinding controller from [21]. (c) represent the finite-time anti-unwinding controller from [17].

In Figs 3–8, (a), (b), and (c) represent the proposed controller, the fixed-time anti-unwinding controller from [21], and the finite-time anti-unwinding controller from [17], respectively.

**Fig 9:**
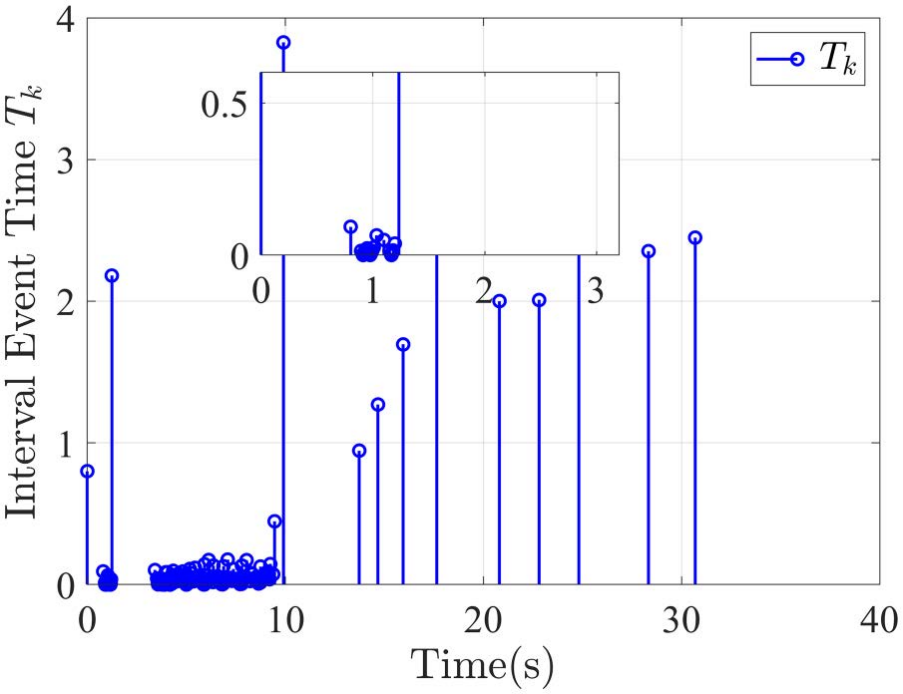
Interevent interval.

The attitude error, angular velocity error, and control torque responses of the proposed controller, the fixed-time anti-unwinding controller presented in [21], and the finite-time anti-unwinding controller proposed in [17] are shown in Figs 3 through 8, respectively. Specifically, Fig 3 illustrates the time response of the scalar part of the attitude quaternion; Fig 4 shows the time response of the vector part of the attitude quaternion; Fig 5 presents the angular velocity response; Fig 6 shows the attitude error response; Fig 7 depicts the angular velocity error response; Fig 8 displays the control torque response; and Fig 9 illustrates the event-triggering interval response.

As shown in Fig 3, the proposed controller (Fig 3(a)) achieves faster convergence to steady state near 1 in 5 seconds than the controllers in Figs 3(b) and 3(c), which indicates stronger anti-unwinding capability. From Figs 4 to 7, it can be seen that the proposed controller drives attitude and velocity errors to zero within 2 seconds, while [21] and [17] require 8 and 20 seconds respectively to convergence. This means that the proposed control scheme achieves faster convergence and better control accuracy than [21] and [17].The time response of the control torque is provided in Fig 8, and the control inputs generated by all three controllers are suitable for practical implementation. Furthermore, as shown in Fig 9, the proposed event-triggered control strategy avoids continuous control signal updates, thereby reducing the communication burden on the spacecraft.

## 5. Conclusions

This paper investigates anti-unwinding attitude tracking control for spacecraft subject to inertia uncertainties and external disturbances. A novel predefined-time sliding mode function is proposed to ensure both predefined-time convergence of the sliding surface and anti-unwinding performance. Unlike most existing works, the proposed method guarantees anti-unwinding behavior not only on the sliding surface but also outside of it. By improving the controller design, chattering is effectively suppressed while preserving both anti-unwinding capability and predefined-time convergence. An event-triggered mechanism is introduced to reduce the communication burden. Finally, simulation results demonstrate the superior anti-unwinding performance and fast convergence of the proposed method compared with existing approaches. Future work will focus on extending predefined-time anti-unwinding control to satellite formation flying scenarios.
